# Integrated transcriptomic analysis of COVID-19 stages and recovery: insights into key gene signatures, immune features, and diagnostic biomarkers through machine learning

**DOI:** 10.3389/fgene.2025.1599867

**Published:** 2025-05-15

**Authors:** Zhiyuan Gong, He An

**Affiliations:** ^1^ School of Medical Technology, Tianjin Medical University, Tianjin, China; ^2^ Department of Clinical Laboratory, Tianjin First Central Hospital, School of Medicine, Nankai University, Tianjin, China

**Keywords:** COVID-19, gene expression profiling, immune system phenomena, random forest, LASSO regression

## Abstract

**Background:**

COVID-19 progression and recovery involve complex gene expression changes and immune dysregulation, but their dynamic alterations remain poorly understood. Current clinical indicators lack precision in distinguishing severe cases, highlighting the need for molecular biomarkers and diagnostic tools.

**Methods:**

Three transcriptomic datasets were analyzed: 1) COVID-19 progression from Healthy, Moderate, Severe, to ICU patients; 2) recovery stages (1, 3, and 6 months) compared to Healthy controls; and 3) COVID-19 ICU versus non-ICU patients. Differential expression analysis, immune cell infiltration estimation, machine learning (LASSO regression and random forest), and functional enrichment were used to identify key genes and molecular mechanisms.

**Results:**

Gene expression analysis revealed dynamic changes during COVID-19 progression. Adaptive immune cells (e.g., B cells and T cells) decreased, while innate immune cells (e.g., monocytes and neutrophils) increased, particularly in ICU patients. Recovery analysis showed significantly reduced adaptive immune cells at 1 month, with partial recovery by 3 and 6 months. Machine learning identified CCR5, CYSLTR1, and KLRG1 as diagnostic biomarkers for distinguishing ICU from non-ICU patients, with AUC values of 0.916, 0.885, and 0.899, respectively.

**Conclusion:**

This study identified CCR5, CYSLTR1, and KLRG1 as efficient diagnostic biomarkers for severe COVID-19 using machine learning and revealed immune regulatory features across COVID-19 progression and recovery.

## 1 Introduction

The global COVID-19 pandemic, caused by the SARS-CoV-2 virus, has led to significant morbidity and mortality worldwide. Despite extensive research, the mechanisms underlying disease progression and recovery remain incompletely understood. Clinical manifestations of COVID-19 vary widely, ranging from asymptomatic cases to severe pneumonia, acute respiratory distress syndrome (ARDS), and death. Severe cases are often associated with dysregulated immune responses, including hyperinflammation and impaired adaptive immunity, which are particularly pronounced in patients requiring intensive care unit (ICU) admission (Chen et al., 2020; Wang et al., 2022; Blanco-Melo et al., 2020). While clinical scores such as the Sequential Organ Failure Assessment (SOFA) and biomarkers like C-reactive protein (CRP) are routinely used, their diagnostic efficacy in distinguishing severe cases remains limited (Herold et al., 2020; Zhou et al., 2020). There is an urgent need for precise molecular markers to improve patient stratification and inform treatment strategies.

Transcriptomic analysis offers a powerful approach to investigate the dynamic gene expression changes associated with disease progression and recovery. Previous studies have demonstrated that transcriptomic signatures can provide insights into the immune landscape, highlighting shifts in immune cell populations and identifying pathways involved in disease pathogenesis (Ou et al., 2024; Cappelletti et al., 2023). Immune profiling, particularly through the analysis of immune cell infiltration, can further delineate the roles of adaptive and innate immune responses in COVID-19. However, few studies have comprehensively examined the gene expression and immune regulation across the full spectrum of COVID-19 stages, from mild cases to ICU admission and through the recovery phase.

Recent advancements in machine learning techniques, such as LASSO regression and random forest algorithms, have enabled the identification of robust diagnostic and prognostic biomarkers from complex transcriptomic datasets (Chen and Ishwaran, 2012; Wang et al., 2024; Fan et al., 2022; Xie et al., 2024). These approaches are particularly effective in handling high-dimensional data, allowing for the identification of key genes associated with severe disease. By integrating transcriptomics with machine learning, we can uncover molecular signatures that are not only diagnostic but also mechanistically linked to disease severity.

In this study, we performed an integrated analysis of three transcriptomic datasets to capture the molecular and immune changes associated with COVID-19 progression and recovery. The first dataset focuses on disease progression across Healthy, Moderate, Severe, and ICU patients; the second examines recovery stages at 1-, 3-, and 6-month post-infection compared to Healthy controls; and the third compares gene expression profiles between ICU and non-ICU patients. Using differential expression analysis, immune infiltration profiling, and machine learning, we identified CCR5, CYSLTR1, and KLRG1 as high-performing diagnostic biomarkers for distinguishing ICU patients. Functional enrichment and immune correlation analyses further revealed the roles of these genes in immune regulation and inflammation, providing novel insights into COVID-19 pathogenesis and recovery mechanisms.

This comprehensive approach highlights the potential of integrating transcriptomic analysis and machine learning to address the critical unmet need for precise diagnostic biomarkers in severe COVID-19. By identifying key molecular signatures, this study contributes to the development of targeted diagnostic and therapeutic strategies, advancing precision medicine for COVID-19 and other infectious diseases.

## 2 Materials and methods

### 2.1 Data sources and processing

**FIGURE 1:**
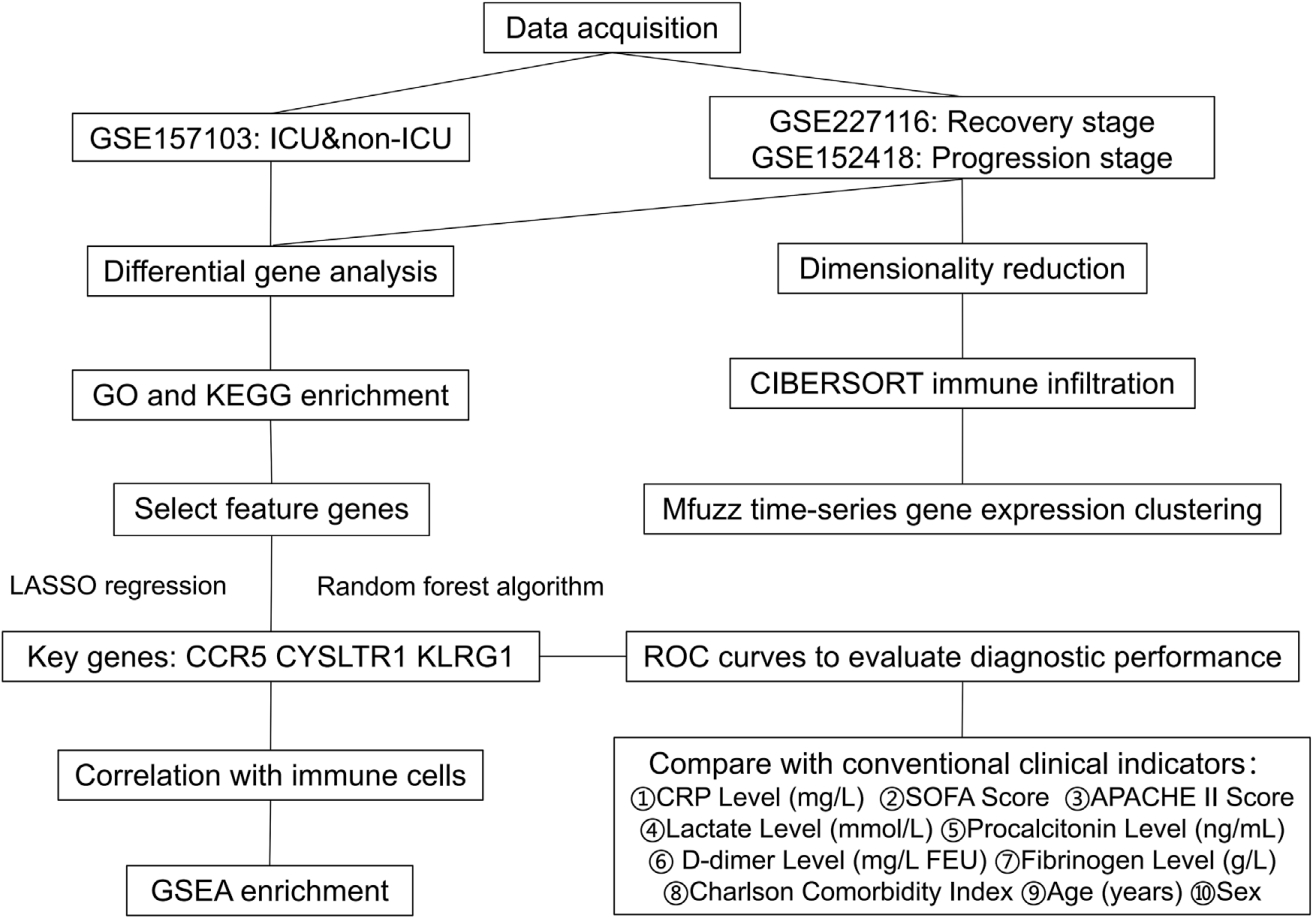
The flow-chart of this study.

We downloaded the gene expression profile from the GEO database (https://www.ncbi.nlm.nih.gov/geo/). The clinical information of the samples included in this study is provided in Table 1, with additional details available in Supplementary Table S1. The gene expression matrix was standardized using R software (version 4.2.2), which included probe annotation, duplicate removal, and log-transformation normalization. Figure 1 Show the process framework of this study.

**TABLE 1:** Information and grouping of the samples.

	Number of samples (included)	Platforms	Status
GSE152418	17	GPL24676 Illumina NovaSeq 6,000	Healthy
	4		Moderate
	8		Severe
	4		ICU
GSE227116	10	GPL16791 Illumina HiSeq 2500	Healthy
	22		1 month hospital discharge
	25		3 months hospital discharge
	18		6 months hospital discharge
GSE157103	50	GPL24676 Illumina NovaSeq 6,000	COVID-19&ICU
	50		COVID-19

### 2.2 t-SNE, PCA, and UMAP analysis

t-SNE (t-distributed Stochastic Neighbor Embedding), PCA (Principal Component Analysis), and UMAP (Uniform Manifold Approximation and Projection) were employed to perform dimensionality reduction and visualization of gene expression data across different stages of COVID-19 progressive and recovery stages. Before analysis, the data were standardized and processed using R software. PCA was used to capture overall variance through principal components, t-SNE emphasized the visualization of local data structures, and UMAP balanced both local and global structures to reveal clustering patterns and group relationships.

### 2.3 Identification of differentially expressed genes (DEGs)

DEGs were identified using the “limma” R package (Ritchie et al., 2015). Pairwise comparisons were conducted across the groups to determine significant DEGs. Adjusted p-values were calculated using the Benjamini–Hochberg method to control for false discovery rates, and genes with an adjusted p-value <0.05 and |log2 fold-change| > 1 were considered significant.

### 2.4 Pattern clustering heatmap

Mfuzz is a clustering algorithm designed for time-series gene expression data (Kumar and M, 2007). Based on the principle of fuzzy clustering, it can handle noise and complexity in gene expression data while identifying groups of genes with similar expression patterns. At its core is the fuzzy C-means clustering algorithm, which allows a gene to belong to multiple cluster centers. The membership of each gene to a cluster center is defined using fuzzy set theory (Kumar and M, 2007). Mfuzz optimizes an objective function to determine the cluster centers and the membership degrees of genes, ensuring that the clustering results better reflect the actual data distribution. Through clustering, it is possible to identify genes with similar expression patterns, providing a foundation for further biological analysis.

### 2.5 Estimation of immune cell infiltration

CIBERSORT is a commonly used computational tool for analyzing immune infiltration from gene expression data (Newman et al., 2015). It employs a deconvolution method to estimate the relative abundances of 22 distinct immune cell types within a heterogeneous cell population. Using the predefined LM22 reference gene expression signature matrix, the algorithm applies support vector regression to provide accurate and reliable assessments of immune cell composition (Newman et al., 2015).

Immune cell infiltration was estimated using the CIBERSORT algorithm, which provides relative proportions of 22 immune cell types based on gene expression data. Statistical comparisons were conducted across groups to identify significant changes in immune cell distributions during disease progression and recovery stages.

### 2.6 Functional and pathway enrichment analysis

Functional annotation and pathway analysis were conducted using the DAVID database (Dennis et al., 2003) (https://david.ncifcrf.gov/home.jsp). Gene Ontology (GO) analysis was categorized into three aspects: molecular function (MF), biological process (BP), and cellular component (CC). Kyoto Encyclopedia of Genes and Genomes (KEGG) pathway analysis was also performed. A significance threshold of P < 0.05 was applied for both GO and KEGG enrichment analyses.

### 2.7 LASSO regression

LASSO (Least Absolute Shrinkage and Selection Operator) regression was applied to reduce the dimensionality of the dataset and identify critical diagnostic genes by penalizing less relevant features. The analysis was performed using the “glmnet” R package with the following steps:

Data Preparation:

• Gene expression data was standardized to ensure comparability across genes.
• Sample labels were binarized to represent ICU and non-ICU groups for classification.

Model Parameters:

• A 10-fold cross-validation approach was used to determine the optimal regularization parameter (lambda) that minimizes the classification error.

Feature Selection:

• LASSO imposes an L1 penalty on the regression coefficients, shrinking the coefficients of less relevant genes to zero.
• Genes with non-zero coefficients after penalization were considered significant features for classification.

### 2.8 Random forest

The random forest algorithm was employed to identify key diagnostic genes associated with COVID-19 severity. Random forest is a supervised machine learning method based on decision trees that evaluates feature importance by measuring how much each feature improves the predictive accuracy of the model. The analysis was performed using the “randomForest” R package with the following steps:

Data Preparation:

• Input features consisted of the expression levels of all genes identified in the dataset.
• Sample labels corresponded to COVID-19 severity (ICU and non-ICU groups).

Model Parameters:

• The number of decision trees (ntree) was set to 500 to ensure model stability.
• The number of features considered at each split (mtry) was optimized using cross-validation.

Feature Importance:

• Feature importance was calculated based on the Mean Decrease Gini index, which measures the contribution of each gene to the model’s overall classification performance.
• Genes with the highest importance scores were considered potential biomarkers.

### 2.9 ROC curve analysis

Receiver Operating Characteristic (ROC) curves were used to evaluate the diagnostic performance of selected genes (e.g., CCR5, CYSLTR1, KLRG1) for distinguishing ICU and non-ICU COVID-19 patients. Logistic regression models were applied to compute predicted probabilities, which were then used to generate ROC curves. The area under the curve (AUC) was calculated to assess diagnostic accuracy. The performance of the selected genes was compared with traditional clinical indicators (e.g., CRP and SOFA scores) to validate their superior diagnostic capability.

### 2.10 Immune correlation analysis

Immune correlation analysis was conducted to investigate the relationships between key genes and immune cell subsets estimated using the CIBERSORT algorithm. Based on gene expression data and immune cell infiltration scores, Pearson correlation coefficients were calculated to evaluate the strength of associations between genes and immune cell types. This analysis identified potential roles of the genes in regulating specific immune cell functions. Correlation heatmaps and scatter plots were used to visually present the significant associations between key genes (e.g., CCR5, CYSLTR1, and KLRG1) and immune cell subsets.

### 2.11 Data visualization and statistical analysis

The data visualization in this study was performed using SangerBox 3.0 (http://vip.sangerbox.com/home.html), Weishengxin (http://www.bioinformatics.com.cn/), and R software. All statistical analyses were performed in R software (version 4.2.2). Statistical significance was determined using Student’s t-test or Wilcoxon rank-sum test for pairwise comparisons, and one-way ANOVA for multiple groups. Correlations between gene expression and immune cell proportions were evaluated using Pearson correlation coefficients, as appropriate.

## 3 Results

### 3.1 Gene expression patterns across healthy, moderate, severe, and ICU groups

**FIGURE 2:**
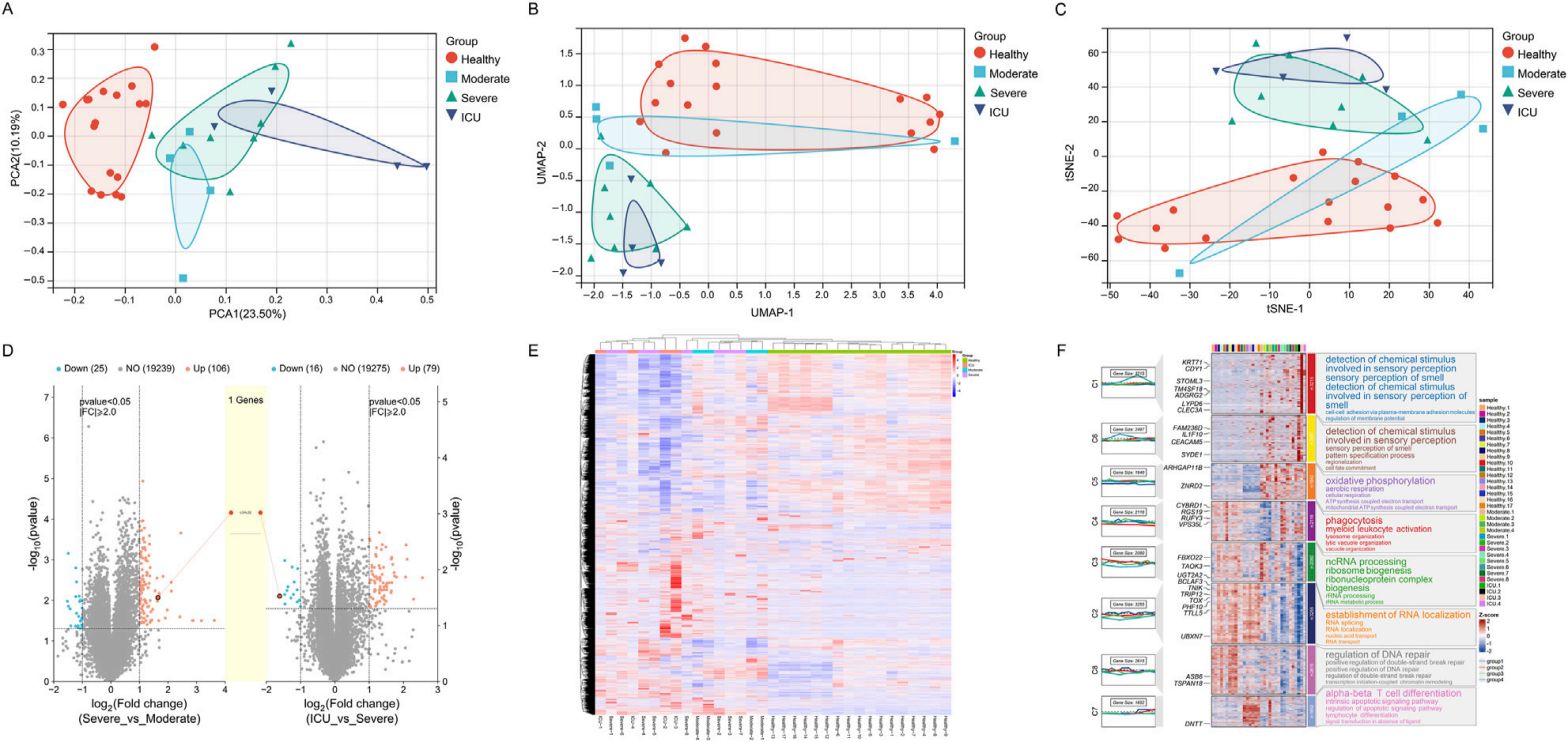
Gene expression patterns across different groups (Healthy, Moderate, Severe, ICU). **(A)** PCA plot showing gene expression distribution and separation among groups. **(B)** UMAP plot illustrating gene expression similarity and divergence across groups. **(C)** t-SNE plot highlighting gene expression clustering and group distinctions. **(D)** Volcano plots depicting differentially expressed genes and a progressively upregulated gene (LGALS2). **(E)** Heatmap of gene expression differences across groups with hierarchical clustering. **(F)** Module heatmap with GO annotations representing group-specific gene expression and associated biological functions.

Through dimensionality reduction analyses (PCA, UMAP, and t-SNE), significant differences in gene expression profiles among COVID-19 patient groups (Healthy, Moderate, Severe, ICU) were revealed. The Healthy group samples formed a tightly clustered group in the reduced dimensional space, distinctly separated from other groups, demonstrating consistent and unique gene expression characteristics. Moderate group samples diverged from the Healthy group, forming an independent cluster while showing proximity to the Severe group, reflecting transitional features in their gene expression profiles. Severe group samples further separated from the Moderate group but exhibited partial overlap with the ICU group, indicating similarities in certain gene expression characteristics. ICU group samples were the most dispersed, showing high heterogeneity, yet remained distinctly separated from other groups, highlighting their unique gene expression profiles (Figures 2A–C). Using the limma R package for differential gene expression analysis, it was found that the Severe group compared to the Moderate group had 106 significantly upregulated genes and 25 significantly downregulated genes. Similarly, the ICU group compared to the Severe group showed 79 significantly upregulated genes and 16 significantly downregulated genes. The central shared gene, LGALS2, exhibited a progressively increasing expression trend, with its expression levels consistently rising from Moderate to Severe and further from Severe to ICU (Figure 2D). This upward trajectory suggests that LGALS2 may play a critical role in the dynamic regulation of disease progression. Its increasing expression correlates with worsening disease severity, indicating its involvement in key pathological processes and highlighting it as a potential target for further investigation. Hierarchical clustering reveals the differences in gene expression between the Healthy group and the other groups (Moderate, Severe, ICU). The distinct colors represent the gene expression characteristics of each group. The clustering results primarily highlight the clear separation of gene expression profiles between the Healthy group and the disease groups (Figure 2E).

Figure 2F illustrates the gene expression changes in COVID-19 patients across different recovery stages, from a healthy state to disease progression leading to intensive care unit (ICU) admission. Through GO functional annotations, it reveals the key biological processes at each stage. The expression changes in gene modules (C1 to C8) display a marked temporal pattern, clearly reflecting the dynamic regulation of biological functions during disease progression. As the disease worsens, gene activation becomes evident in module C4 during the moderate stage, representing the initiation of the innate immune system. The functional annotations of this module highlight processes such as phagocytosis, myeloid leukocyte activation, and lysosomal function, indicating the activation of immune cells to eliminate pathogens triggered by infection. With further disease progression to the severe stage, module C5 shows a significant increase in expression. Its functional annotations focus on oxidative phosphorylation, mitochondrial ATP production, and the electron transport chain, highlighting the central role of metabolic reprogramming at this stage. The increased metabolic demand reflects the need for enhanced mitochondrial function to meet high energy consumption. Concurrently, module C3 displays elevated expression, indicating the enhancement of processes like ncRNA processing, ribosome biogenesis, and RNA metabolism. These changes suggest widespread transcription and protein synthesis activity, likely supporting the functions of immune cells and infected cells during this stage. In ICU patients, module C7 shows a striking upregulation, predominantly involving processes such as alpha-beta T cell differentiation, lymphocyte differentiation, and signal transduction. This reflects the robust activation of the adaptive immune system. At this stage, T cell remodeling likely plays a critical role in responding to viral infection and mitigating uncontrolled inflammation. Another notable change is observed in module C2, whose functional annotations include RNA localization and RNA splicing, suggesting enhanced post-transcriptional regulation, potentially to maintain cellular homeostasis. Module C8 also shows increased activity related to DNA repair and double-strand break repair, indicating significant DNA damage and stress response in severe cases.

### 3.2 Gene expression patterns across 1-month, 3-month, 6-month post-discharge, and healthy groups

**FIGURE 3:**
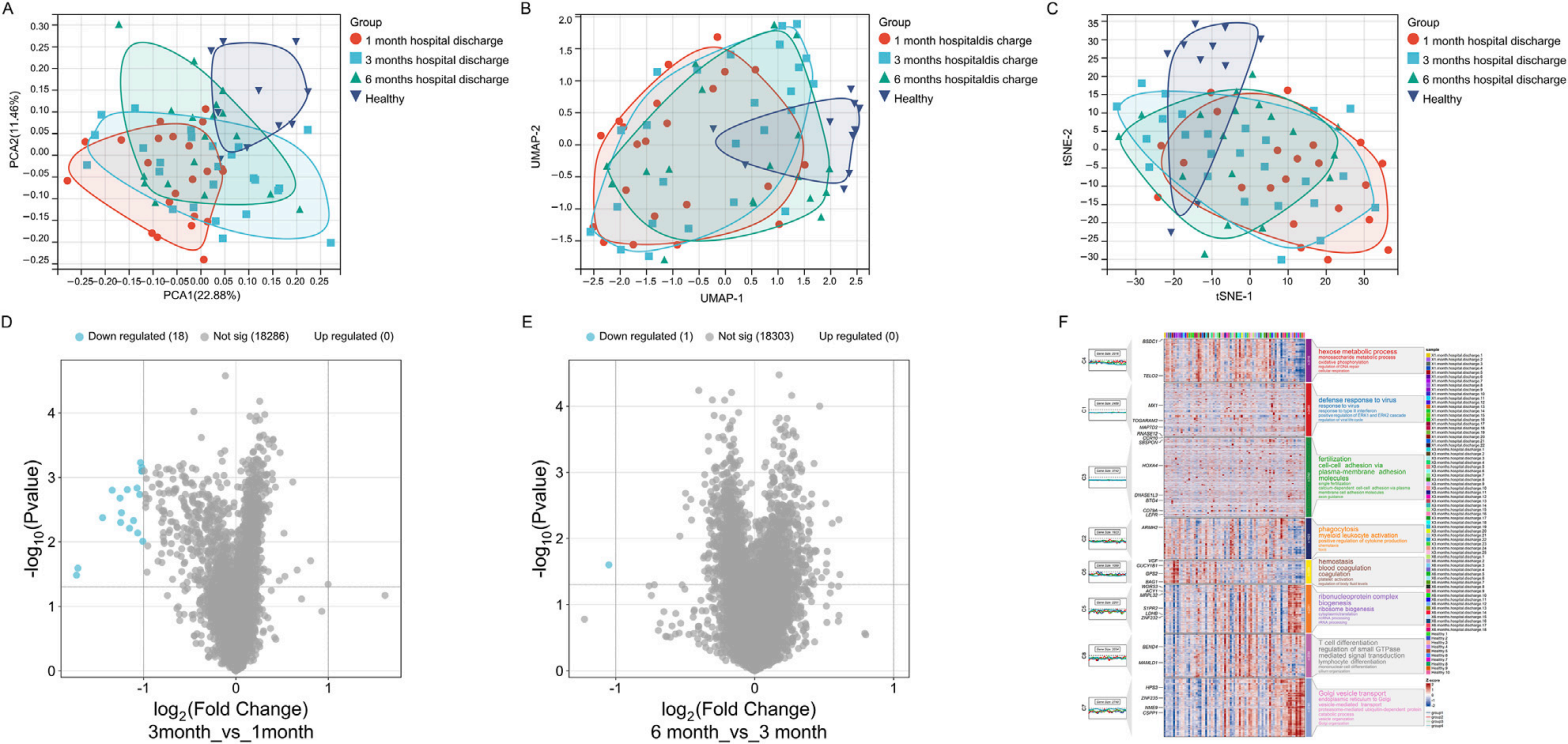
Gene expression patterns across different recovery stages (1-month, 3-month, 6-month post-discharge, Healthy). **(A)** PCA plot showing gene expression distribution and separation. **(B)** UMAP plot illustrating gene expression similarity and divergence. **(C)** t-SNE plot highlighting gene expression clustering and group distinctions. **(D)** Volcano plot showing differentially expressed genes between 3-month and 1-month groups. **(E)** Volcano plot showing differentially expressed genes between 6-month and 3-month groups. **(F)** Module heatmap with GO annotations representing group-specific gene expression and associated biological functions.

The dimensionality reduction plots (PCA, UMAP, t-SNE) reveal significant differences in gene expression patterns among the 1-month, 3-month, and 6-month post-discharge groups compared to the healthy group. The 1-month group is distinctly separated from the healthy group, indicating that early post-discharge patients still exhibit substantial differences in gene expression compared to healthy individuals. The overlap between the 3-month and 6-month groups suggests a gradual recovery of gene expression patterns, though they have not fully returned to the healthy state. The healthy group displays a relatively independent distribution, further emphasizing the significant differences in gene expression between healthy individuals and all post-discharge groups (Figures 3A–C). Volcano plots (Figures 3D,E) compare gene expression differences between the 3-month and 1-month post-discharge groups and between the 6-month and 3-month post-discharge groups. The results show no significantly upregulated genes in either comparison, with only a small number of significantly downregulated genes (18 genes for 3-month vs. 1-month and 1 gene for 6-month vs. 3-month). Overall, the gene expression differences between these groups are minimal, with no notable significance, indicating that gene expression tends to stabilize over these time points.

Although differential gene expression during the recovery period is not highly significant, the gene expression patterns still reveal certain intergroup differences: In the 1-month post-discharge samples, C1 and C2 modules were significantly upregulated, with functional annotations mainly related to virus defense response, type I interferon response, and phagocytosis. These functions indicate that the immune system of early post-discharge patients remains active, potentially in response to residual viral or inflammatory signals. Additionally, the upregulation of the C4 module reflects enhanced oxidative phosphorylation and metabolic activity, suggesting that cellular metabolic functions might be significantly impacted during the early stage of recovery (Figure 3F). At the 3-month stage, the C6 module showed higher expression levels, with functional annotations focusing on coagulation and platelet activation, indicating that the blood system might be gradually returning to normal, though slight coagulation abnormalities might still persist. Moreover, the C3 module, associated with cell adhesion and fertilization-related pathways, suggests further recovery of intercellular signaling and structural regulation. Compared to the 1-month group, the expression of immune-related modules (e.g., C1 and C2) decreased, showing that immune activity was gradually stabilizing but had not yet fully returned to a healthy state (Figure 3F). In the 6-month post-discharge samples, the C5 module (related to ribosome biogenesis and RNA metabolism) and the C8 module (associated with T cell differentiation and immune signal transduction) were significantly upregulated, indicating the importance of transcriptional and adaptive immune system activity at this stage. Meanwhile, the upregulation of the C7 module, involving genes related to Golgi transport and protein degradation, reflects that organelle remodeling and metabolic regulation remain active during the 6-month stage. Although gene expression at this stage is closer to the healthy group, certain immune and metabolic pathways still exhibit differences, suggesting that the recovery process is not yet complete (Figure 3F). Additionally, in healthy samples, gene modules (e.g., C3 and C6) exhibit stable expression, with functional annotations focusing on intercellular communication and coagulation regulation, indicating that gene expression in the healthy state primarily supports basal metabolism and homeostasis, without significant immune or stress signals (Figure 3F).

### 3.3 Immune infiltration analysis across COVID-19 severity stages and recovery phases

**FIGURE 4:**
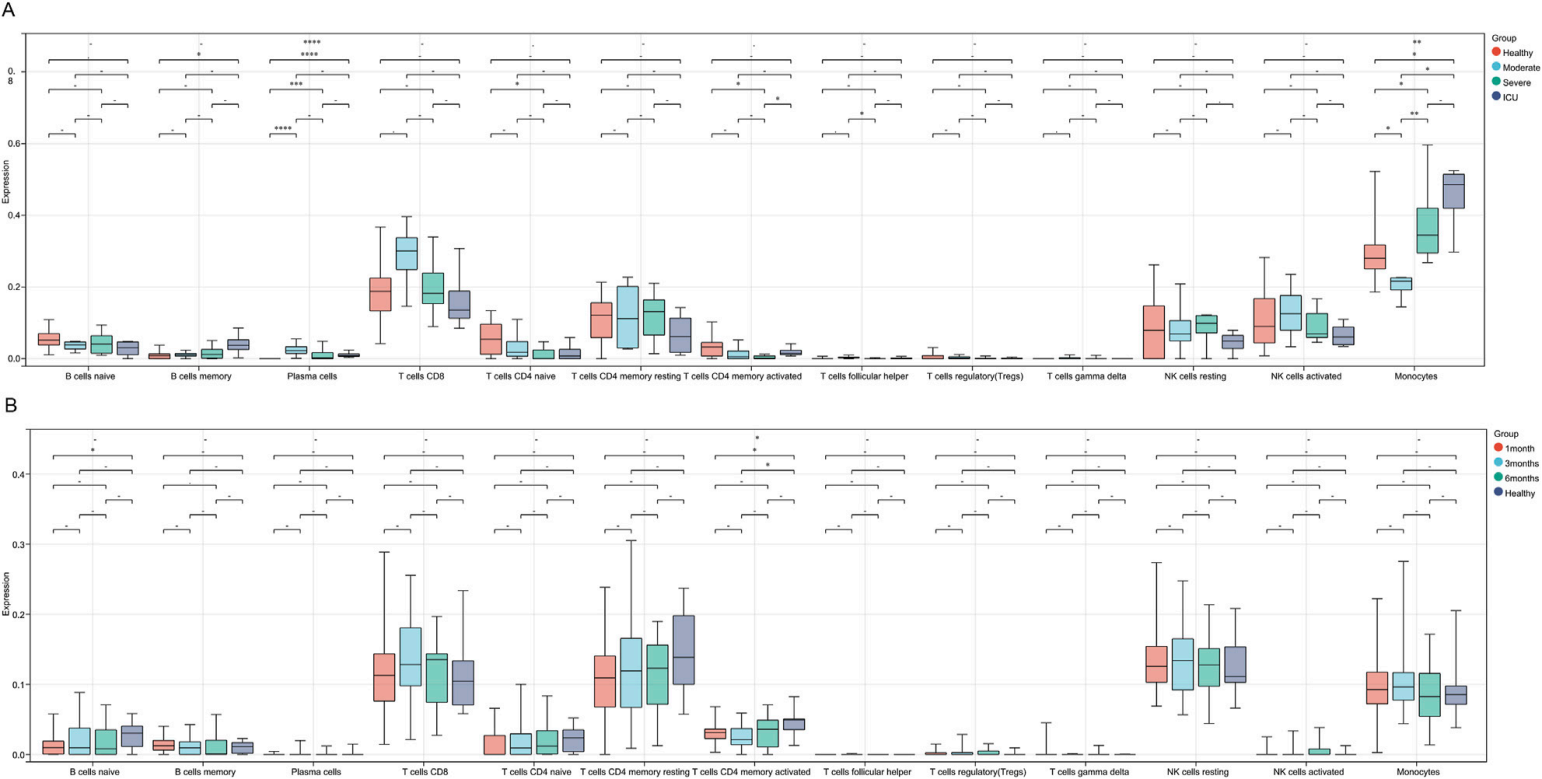
Immune cell infiltration differences across COVID-19 severity stages and recovery phases. **(A)** Boxplots showing immune cell infiltration levels across Healthy, Moderate, Severe, and ICU groups. **(B)** Boxplots illustrating immune cell infiltration levels across 1-month, 3-month, 6-month recovery groups, and Healthy. Significant differences are marked with asterisks (* for P < 0.05 and ** for P < 0.01), while a dash (−) indicates P > 0.05.

Firstly, we performed immune infiltration analysis on samples from COVID-19 patient groups, and the results revealed differences in the expression levels and significance among different immune cell subsets across the Healthy, Moderate, Severe, and ICU groups. B cells memory and Plasma cells showed significantly higher expression in the Healthy group compared to other groups, with the remaining three groups (Moderate, Severe, ICU) exhibiting a progressive decrease as disease severity increased. T cells CD4 naive displayed a gradual decline from the Healthy group to the ICU group, while T cells CD4 memory activated decreased from the Healthy to Severe stages but showed a slight recovery in the ICU group, though still lower than in the Healthy group. Monocytes showed significant differences between groups, with levels progressively increasing from the Moderate group to the Severe and ICU groups, all of which were higher than in the Healthy group. These findings suggest that the progression of COVID-19 suppresses adaptive immunity, as evidenced by the reduced levels of B and T cells, while the marked increase in monocytes reflects enhanced innate immune and inflammatory responses during disease progression (Figure 4A).

Compared to COVID-19 patients at different severity stages, the differences in immune cell infiltration across various recovery stages are generally small, with significant changes observed only in a few subsets. Specifically, the expression level of B cells naive in the 1-month recovery group was significantly lower than that in the Healthy group, indicating a potential insufficiency in adaptive immune function during the early recovery phase. In the 3-month recovery group, the expression level of T cells CD4 memory activated was also significantly lower than that in the Healthy group, suggesting that the functional recovery of activated memory CD4^+^ T cells might still be incomplete during the mid-recovery phase. Additionally, there were certain intergroup differences across recovery stages (Figure 4B).

### 3.4 Differential expression and enrichment analysis between COVID-19 ICU and non-ICU groups

**FIGURE 5:**
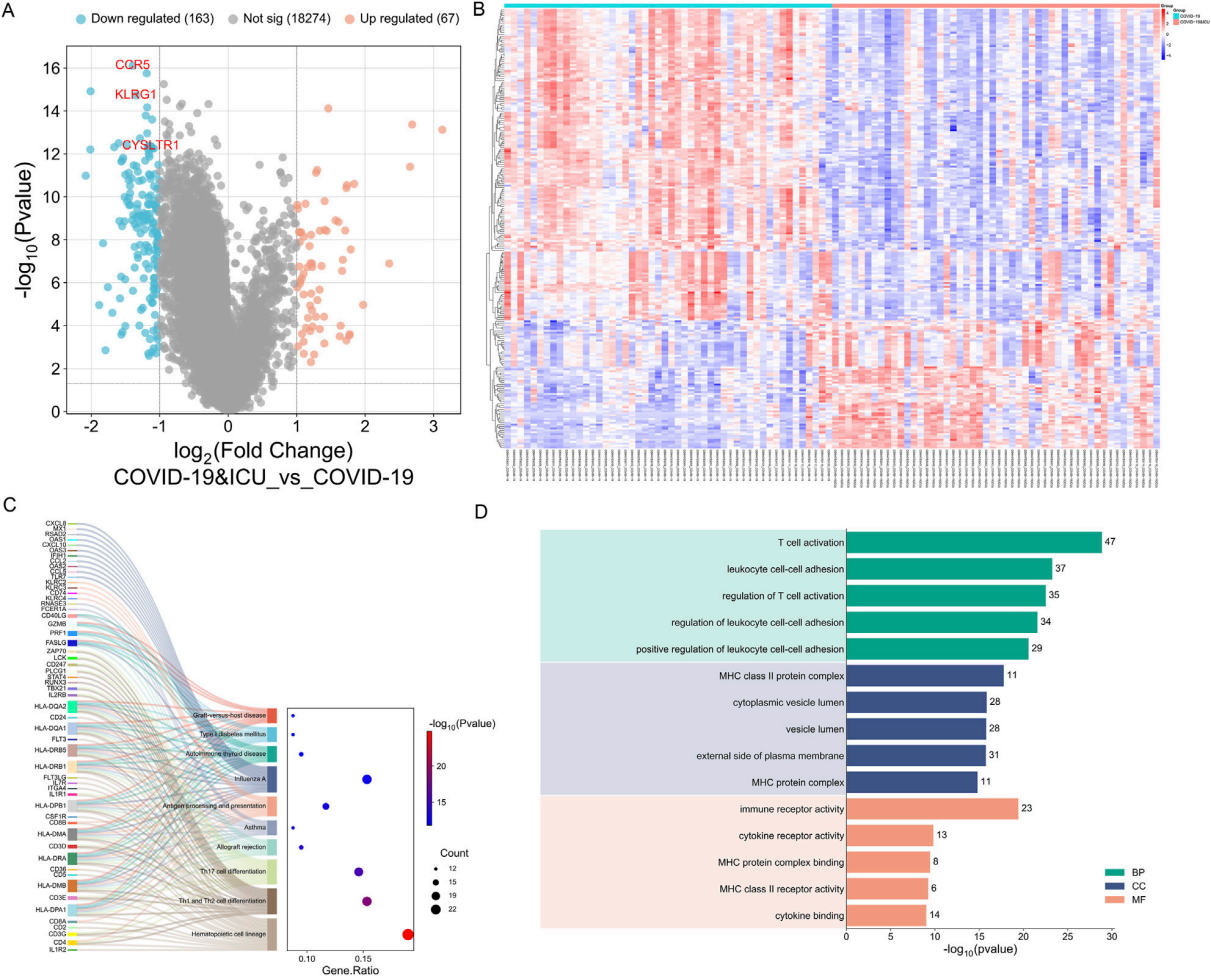
Differential expression and enrichment analysis between COVID-19 ICU and non-ICU groups. **(A)** Volcano plot showing significantly upregulated (red) and downregulated (blue) genes in the ICU group, with key genes (CCR5, KLRG1, CYSLTR1) labeled. **(B)** Heatmap of 230 DEGs highlighting distinct expression patterns between ICU and non-ICU groups. **(C)** KEGG enrichment analysis linking DEGs to pathway. **(D)** GO enrichment analysis categorizing DEGs into BP, CC, and MF.

To further investigate the differences between COVID-19 patients with varying severity levels, we utilized the GSE157103 dataset to analyze gene expression differences between the COVID-19 ICU group and the non-ICU COVID-19 group. Differential expression analysis using the limma R package revealed that, compared to the non-ICU group, the COVID-19 ICU group exhibited 67 upregulated genes and 163 downregulated genes (Figures 5A,B).

Subsequently, GO and KEGG enrichment analyses were performed on the 230 differentially expressed genes. KEGG enrichment analysis revealed that the differentially expressed genes between the COVID-19 ICU group and the non-ICU group were primarily enriched in immune-related pathways, including hematopoietic cell lineage, Th1 and Th2 cell differentiation, Th17 cell differentiation, and antigen processing and presentation. These pathways indicate significant alterations in immune cell differentiation, T cell function regulation, and antigen presentation mechanisms in severe COVID-19 patients. Additionally, the differentially expressed genes were enriched in pathways related to autoimmunity and inflammation, such as type 1 diabetes mellitus, autoimmune thyroid disease, and asthma, suggesting that severe COVID-19 may be accompanied by autoimmune-like responses. Furthermore, the enrichment of pathways such as allograft rejection and graft-versus-host disease further reflects the characteristics of immune dysregulation during the severe stages of COVID-19 (Figure 5C). Overall, these findings uncover the complex mechanisms of immune dysfunction in severe COVID-19 patients and provide important insights for further research and therapeutic interventions.

The GO enrichment analysis revealed that differentially expressed genes were primarily associated with Biological Processes (BP), Cellular Components (CC), and Molecular Functions (MF). In Biological Processes, significant enrichment was observed in pathways such as T cell activation, leukocyte cell-cell adhesion, and regulation of T cell activation, highlighting the critical role of T cell function and immune cell interactions in the progression of COVID-19. For Cellular Components, the genes were significantly enriched in MHC class II protein complex, cytoplasmic vesicle lumen, and external side of plasma membrane, suggesting the importance of antigen presentation and intracellular transport in immune regulation. In Molecular Functions, enriched terms included immune receptor activity, cytokine receptor activity, and MHC class II receptor activity, reflecting notable changes in immune signaling and cytokine regulation (Figure 5D). These findings indicate that the differentially expressed genes associated with COVID-19 play essential roles in immune response and regulation, providing valuable insights into the disease mechanisms.

### 3.5 Immune infiltration analysis between COVID-19 ICU and non-ICU groups

**FIGURE 6:**
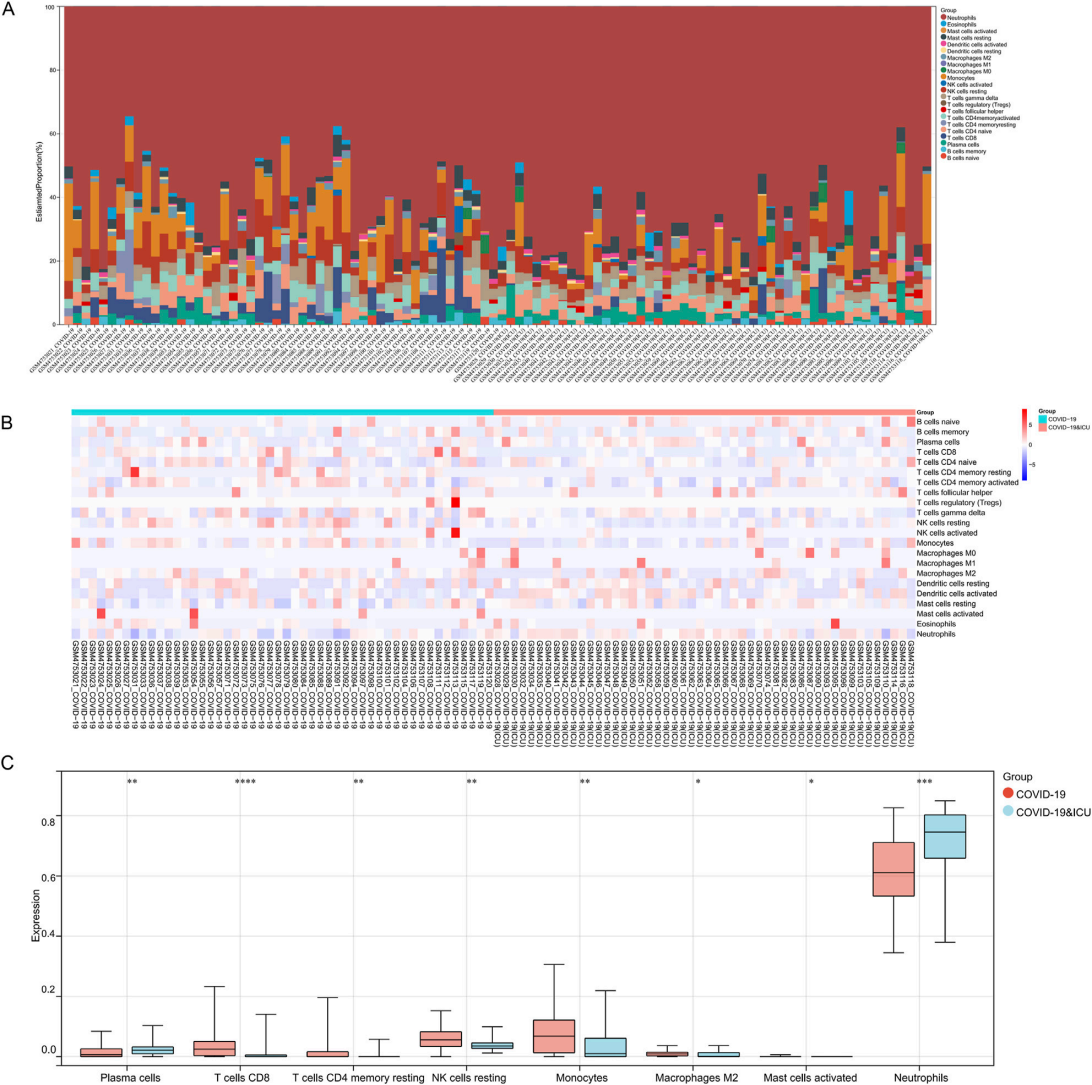
Immune infiltration analysis between COVID-19 ICU and non-ICU groups. **(A)** Stacked bar plot showing the proportions of 22 immune cell types across samples in the COVID-19 ICU and non-ICU groups. **(B)** Heatmap of immune cell infiltration scores, highlighting differences in immune cell distribution between the two groups. **(C)** Boxplots comparing significantly different immune cell types between the COVID-19 ICU and non-ICU groups, including plasma cells, T cells CD8, T cells CD4 memory resting, NK cells resting, monocytes, macrophages M2, mast cells activated, and neutrophils. Significant differences are marked with asterisks (* for P < 0.05 and ** for P < 0.01), while a dash (−) indicates P > 0.05.

In the immune infiltration analysis of the COVID-19 ICU and non-ICU groups, significant differences were observed in the distribution of several immune cell subsets, highlighting immune system imbalance in severe patients. The stacked bar plot illustrates the proportions of 22 immune cell types in samples from the ICU and non-ICU groups, revealing certain differences in immune cell proportions between the two groups (Figure 6A). The heatmap further highlights significant differences in immune infiltration patterns between the ICU and non-ICU groups (Figure 6B). Boxplot analysis showed significant differences in several immune cell subsets between the two groups. Monocytes, neutrophils, M2 macrophages, and activated mast cells were significantly higher in the ICU group than in the non-ICU group, reflecting abnormal activation of innate immune cells in severe patients, which may be closely related to excessive inflammatory responses. Conversely, plasma cells, CD8^+^ T cells, CD4^+^ memory T cells (resting), and resting NK cells were significantly lower in the ICU group compared to the non-ICU group, suggesting suppression of adaptive immune function. Notably, the reduced activity of T cells and NK cells might impair the ability to clear the virus (Figure 6C). These findings suggest that excessive activation of innate immunity and suppression of adaptive immunity may be key features of immune imbalance in ICU patients with COVID-19, providing critical insights for understanding the pathological mechanisms of severe COVID-19.

### 3.6 Feature gene selection for COVID-19 ICU patients using LASSO regression and random forest algorithm

**FIGURE 7:**
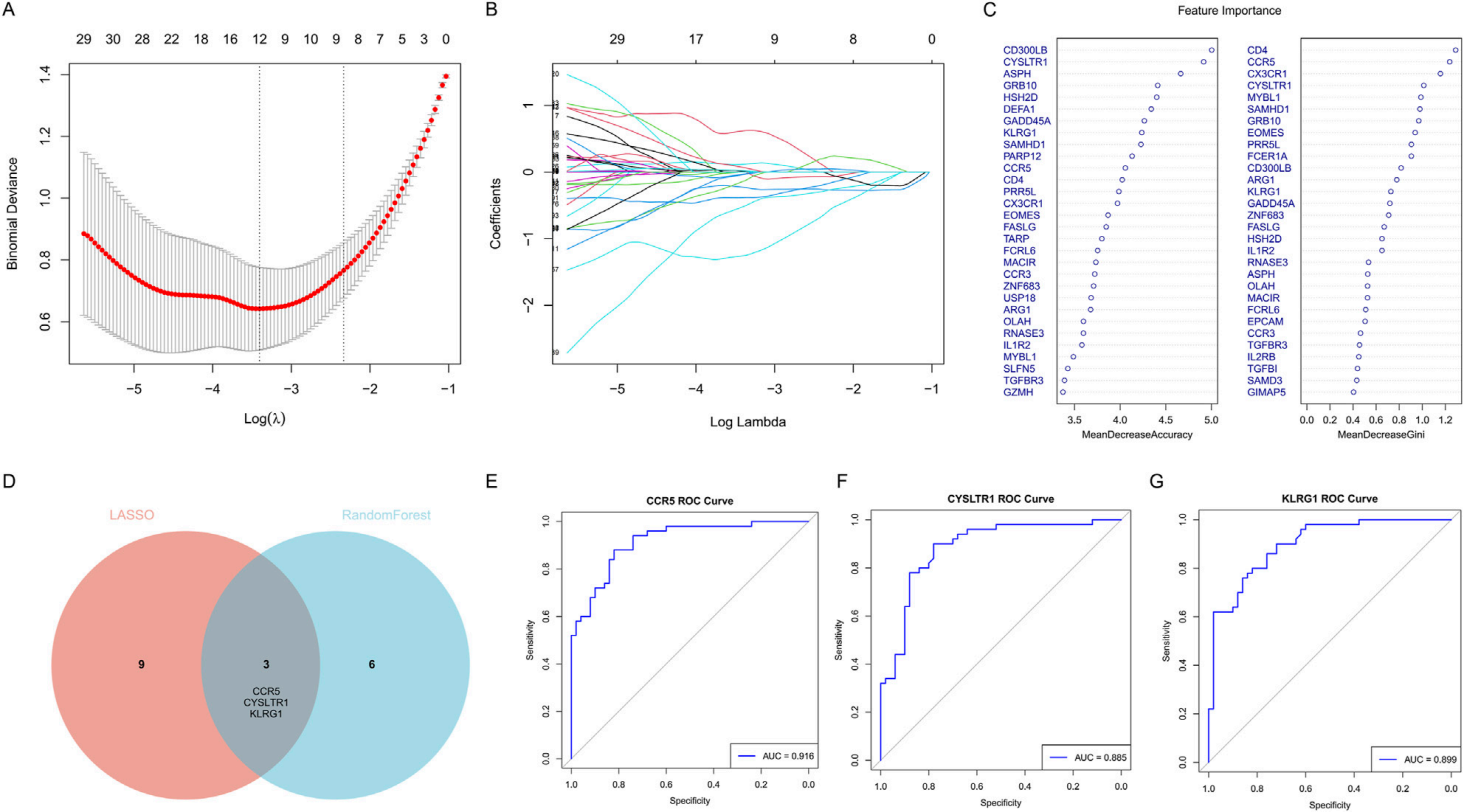
Identification of key feature genes for COVID-19 ICU patients using LASSO regression and random forest algorithms. **(A)** Cross-validation plot for the LASSO regression model to identify optimal lambda values. **(B)** LASSO coefficient profiles of candidate genes as a function of the regularization parameter (log lambda). **(C)** Feature importance rankings from the random forest algorithm, displayed by Mean Decrease Accuracy and Mean Decrease Gini. **(D)** Venn diagram showing the overlap of selected genes from LASSO regression and random forest algorithms. **(E–G)** ROC curves showing the diagnostic performance of the three key genes.

To identify key genes significantly associated with COVID-19 ICU patients, we employed LASSO regression and random forest algorithms. LASSO regression, utilizing L1 regularization, effectively eliminated redundant variables and identified a small number of highly predictive feature genes (Figures 7A,B). The random forest algorithm further validated and selected relevant genes by evaluating their importance (Figure 7C). Ultimately, the intersection of the two methods identified three key genes: CCR5, CYSLTR1, and KLRG1 (Figure 7D).

Subsequently, the diagnostic performance of these three genes was evaluated using ROC curves (Figures 7E–G). CCR5 achieved an AUC value of 0.916, CYSLTR1 an AUC value of 0.885, and KLRG1 an AUC value of 0.899, all demonstrating excellent predictive performance. These findings indicate that CCR5, CYSLTR1, and KLRG1 are important feature genes for COVID-19 ICU patients, providing critical insights for further research and clinical diagnosis.

### 3.7 Diagnostic value and immune regulatory roles of CCR5, CYSLTR1, and KLRG1 in COVID-19 ICU patients

**FIGURE 8:**
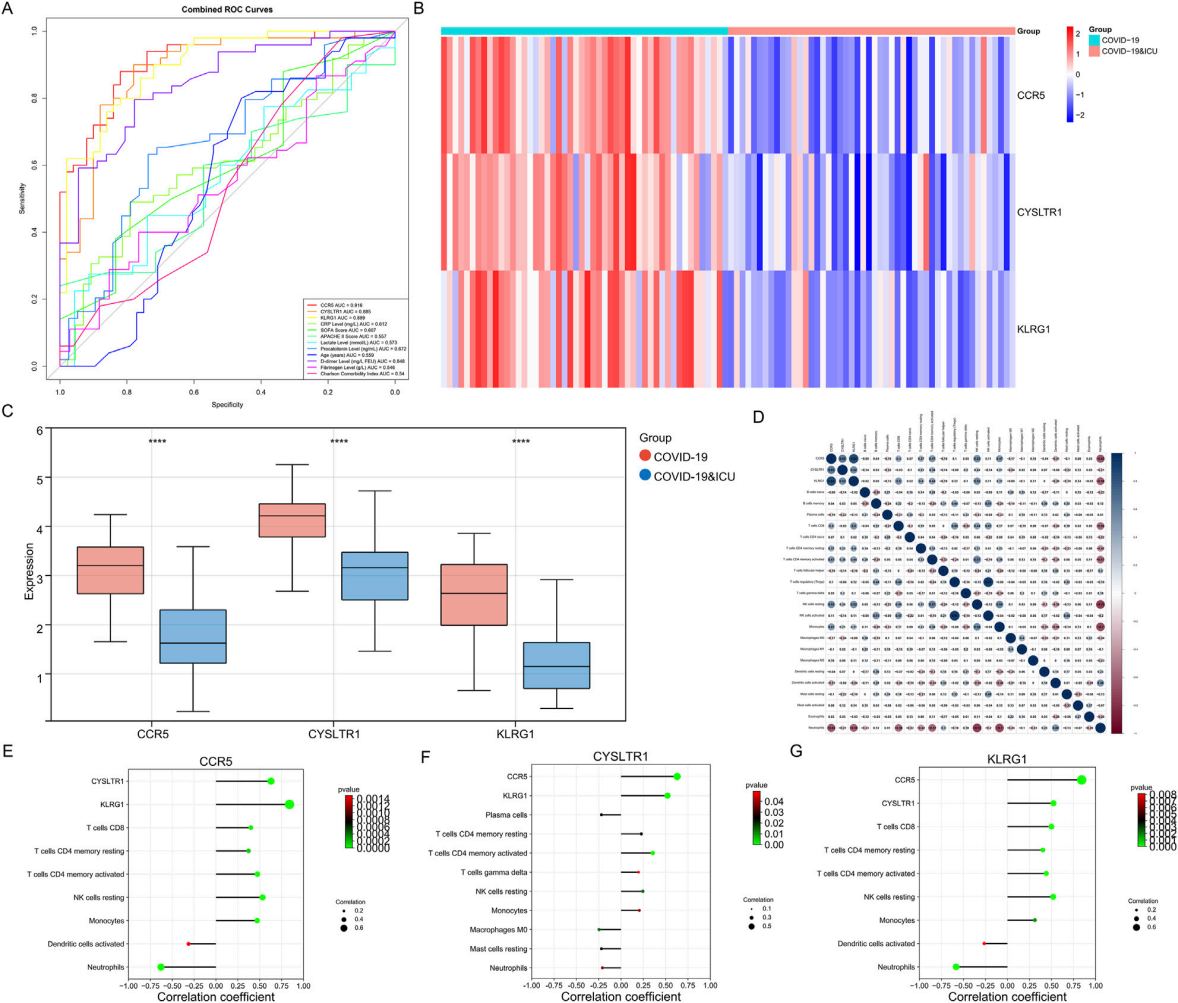
Diagnostic performance, expression patterns, and immune correlations of CCR5, CYSLTR1, and KLRG1. **(A)** ROC curves for CCR5, CYSLTR1, and KLRG1 compared to conventional clinical indicators (e.g., CRP, SOFA, APACHE II scores). **(B)** Heatmap showing differential expression of CCR5, CYSLTR1, and KLRG1 in COVID-19 ICU and non-ICU groups. **(C)** Boxplots displaying significantly lower expression levels of CCR5, CYSLTR1, and KLRG1 in COVID-19 ICU patients compared to non-ICU patients. **(D)** Correlation matrix illustrating relationships between CCR5, CYSLTR1, KLRG1, and immune cell infiltration scores. **(E–G)** Correlation plots showing specific relationships between CCR5 **(E)**, CYSLTR1 **(F)**, and KLRG1 **(G)** with immune cell subtypes.

Clinical information from 100 patients with COVID-19 stratified by ICU admission status is shown in Table 2. Among the 100 COVID-19 patients included in this study, ICU patients (n = 50) exhibited significant differences in several clinical characteristics compared to non-ICU patients (n = 50). Inflammatory markers such as CRP, procalcitonin, and D-dimer were significantly elevated in ICU patients, indicating a stronger inflammatory response. Additionally, ICU patients had higher SOFA and APACHE II scores, reflecting greater disease severity. While the gender distribution was similar between the two groups, ICU patients were older on average and had slightly higher comorbidity indices. Laboratory parameters such as fibrinogen and lactate levels showed minimal differences, but ICU patients exhibited more pronounced inflammatory and pathological features overall. ROC curve analysis (Figure 8A) demonstrated that CCR5 (AUC = 0.916), CYSLTR1 (AUC = 0.885), and KLRG1 (AUC = 0.899) exhibit superior diagnostic performance in distinguishing COVID-19 ICU patients from non-ICU patients compared to conventional clinical indicators such as C-reactive protein (CRP, AUC = 0.612), SOFA score (AUC = 0.607), APACHE II score (AUC = 0.557), and D-dimer levels (AUC = 0.848). Additionally, other indicators such as age (AUC = 0.559), fibrinogen levels (AUC = 0.546), and Charlson Comorbidity Index (AUC = 0.540) showed weaker diagnostic power. These results suggest that CCR5, CYSLTR1, and KLRG1 have significant advantages in diagnosing severe COVID-19 cases, offering critical molecular biomarkers for accurate patient identification.

**TABLE 2:** Clinical information from 100 patients with COVID-19 of varying disease severity.

	COVID-19 (N = 50)	COVID-19&ICU (N = 50)	Total (N = 100)
CCR5			
Mean ± SD	3.11 ± 0.67	1.71 ± 0.72	2.41 ± 0.99
Median [min-max]	3.20 [1.65,4.24]	1.63 [0.24,3.59]	2.40 [0.24,4.24]
CYSLTR1			
Mean ± SD	4.10 ± 0.60	2.97 ± 0.75	3.53 ± 0.88
Median [min-max]	4.21 [2.68,5.26]	3.16 [1.46,4.72]	3.63 [1.46,5.26]
KLRG1			
Mean ± SD	2.55 ± 0.79	1.20 ± 0.63	1.88 ± 0.98
Median [min-max]	2.64 [0.67,3.86]	1.15 [0.30,2.92]	1.83 [0.30,3.86]
CRP Level (mg/L)			
Mean ± SD	119.82 ± 95.49	158.72 ± 107.97	140.54 ± 103.62
Median [min-max]	114.00 [1.00,430.50]	147.30 [2.80,408.80]	128.20 [1.00,430.50]
SOFA Score			
Mean ± SD	6.50 ± 3.62	8.30 ± 4.05	8.11 ± 4.01
Median [min-max]	7.00 [2.00,12.00]	7.50 [2.00,19.00]	7.00 [2.00,19.00]
APACHE II Score			
Mean ± SD	19.71 ± 5.91	21.58 ± 8.49	21.35 ± 8.19
Median [min-max]	19.00 [11.00,27.00]	21.50 [6.00,43.00]	21.00 [6.00,43.00]
Lactate Level (mmol/L)			
Mean ± SD	1.19 ± 0.53	1.27 ± 0.49	1.24 ± 0.51
Median [min-max]	1.09 [0.65,3.28]	1.20 [0.50,2.85]	1.17 [0.50,3.28]
Procalcitonin Level (ng/mL)			
Mean ± SD	1.71 ± 5.82	4.43 ± 12.89	3.24 ± 10.45
Median [min-max]	0.36 [0.05,36.00]	1.02 [0.05,86.39]	0.57 [0.05,86.39]
Age (years)			
Mean ± SD	58.96 ± 18.40	62.64 ± 13.60	60.84 ± 16.15
Median [min-max]	56.50 [24.00,87.00]	63.00 [21.00,83.00]	62.00 [21.00,87.00]
D-dimer Level (mg/L FEU)			
Mean ± SD	1.98 ± 3.21	18.88 ± 27.52	11.72 ± 22.53
Median [min-max]	0.99 [0.22,15.48]	6.03 [0.59,104.42]	1.79 [0.22,104.42]
Sex			
female	21 (21.00%)	17 (17.00%)	38 (38.00%)
male	29 (29.00%)	33 (33.00%)	62 (62.00%)
Fibrinogen Level (g/L)			
Mean ± SD	563.56 ± 191.72	528.96 ± 201.64	543.85 ± 196.94
Median [min-max]	513.00 [215.00,949.00]	490.00 [140.00,949.00]	513.00 [140.00,949.00]
Charlson Comorbidity Index			
Mean ± SD	3.12 ± 2.46	3.44 ± 2.51	3.28 ± 2.48
Median [min-max]	2.50 [0,8.00]	3.00 [0.0,11.00]	3.00 [0,11.00]

Expression analysis revealed significant differential expression of CCR5, CYSLTR1, and KLRG1 between COVID-19 ICU and non-ICU groups (Figure 8B). These three genes were significantly upregulated in the non-ICU group and downregulated in the ICU group. Boxplot further validated this finding, showing significantly lower expression levels of the three genes in the ICU group compared to the non-ICU group (Figure 8C). These results suggest that the expression levels of these genes may negatively correlate with disease severity, further supporting their potential as molecular biomarkers for severe COVID-19. Correlation analysis highlighted the associations between these genes and immune cell infiltration, shedding light on their possible mechanisms of action (Figure 8D). Specifically, CCR5 was positively correlated with several T cell subtypes, including CD8^+^ T cells and CD4^+^ memory T cells (both resting and activated), highlighting its essential role in adaptive immunity. Additionally, CCR5 was negatively correlated with neutrophils and monocytes, suggesting its potential involvement in suppressing excessive innate immune activation to maintain immune balance (Figure 8E). CYSLTR1 showed significant positive correlations with monocytes and resting NK cells, indicating its role in regulating inflammatory responses and immune cell differentiation (Figure 8F). KLRG1 was highly positively correlated with NK cells (both resting and activated), reflecting its critical role in immune surveillance and cytotoxic responses mediated by NK cells. Additionally, KLRG1 showed positive correlations with monocytes and resting dendritic cells, suggesting its involvement in the regulation of innate immune cell functions (Figure 8G). In summary, CCR5, CYSLTR1, and KLRG1 not only demonstrate significant diagnostic value for identifying severe COVID-19 patients but also play critical roles in modulating immune cell activation, differentiation, and function. These findings provide new insights into the immune regulatory mechanisms in severe COVID-19 and offer promising molecular targets for diagnostic and therapeutic strategies.

### 3.8 GSEA enrichment analysis of three key diagnostic genes

To comprehensively understand the functional implications of the three key diagnostic genes (CCR5, CYSLTR1, and KLRG1), GSEA was performed based on KEGG and GO datasets.

**FIGURE 9:**
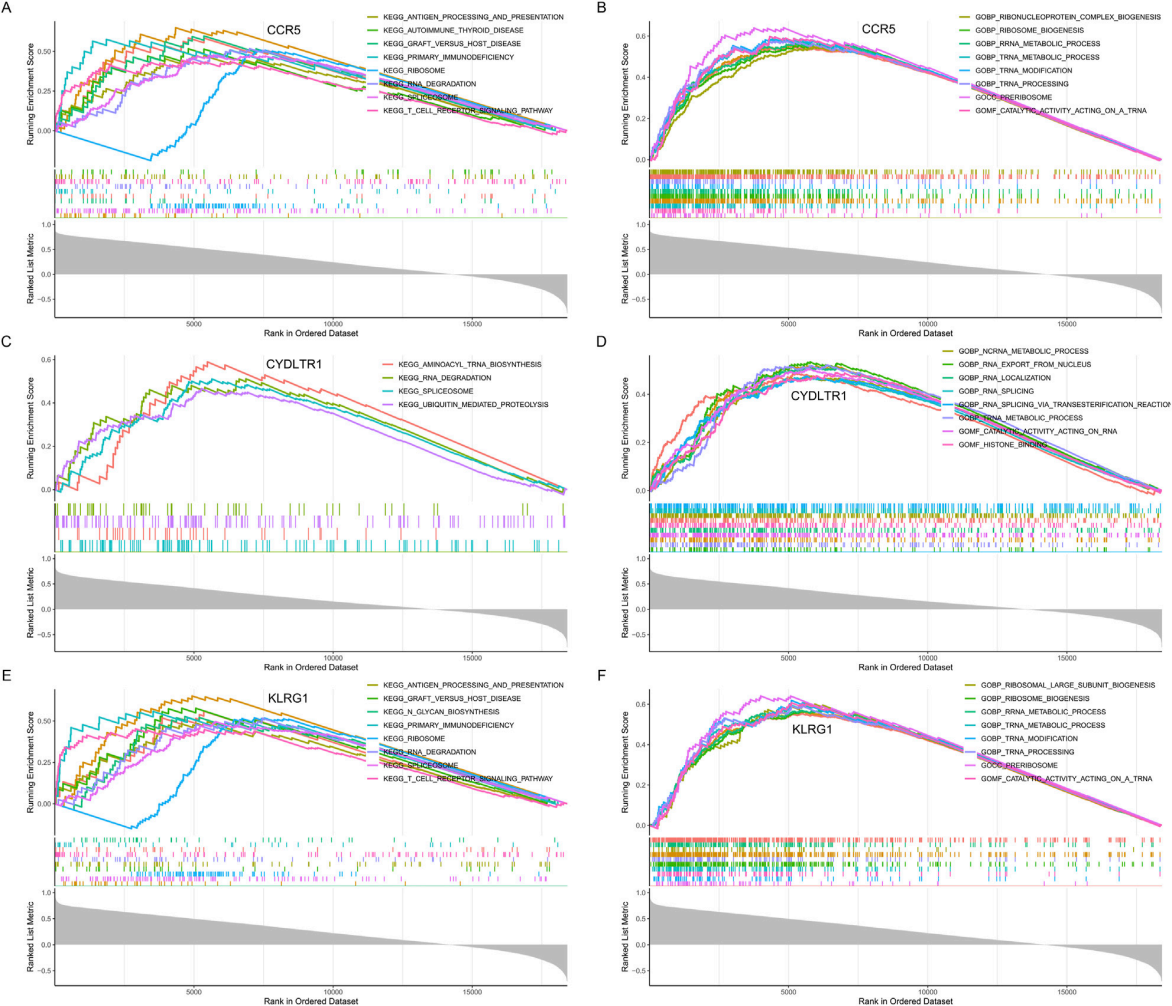
GSEA enrichment analysis for CCR5, CYSLTR1, and KLRG1. **(A)** KEGG pathway enrichment analysis for CCR5. **(B)** GO enrichment analysis for CCR5. **(C)** KEGG pathway enrichment analysis for CYSLTR1. **(D)** GO enrichment analysis for CYSLTR1. **(E)** KEGG pathway enrichment analysis for KLRG1. **(F)** GO enrichment analysis for KLRG1.

CCR5 was predominantly enriched in immune-related pathways. KEGG analysis (Figure 9A) identified antigen processing and presentation, T cell receptor signaling, and primary immunodeficiency as significantly enriched pathways, underscoring CCR5’s pivotal role in adaptive immune regulation. GO enrichment (Figure 9B) highlighted its involvement in ribosome biogenesis, RNA metabolic processes, and cellular metabolic pathways, suggesting its contribution to maintaining cellular activity and immune function.

CYSLTR1 demonstrated enrichment in both metabolic and regulatory pathways. KEGG analysis (Figure 9C) indicated significant associations with aminoacyl-tRNA biosynthesis, RNA degradation, and ubiquitin-mediated proteolysis, revealing its role in protein synthesis and degradation. Similarly, GO enrichment (Figure 9D) identified key processes such as transcription regulation, nuclear transport, and broader metabolic activities, pointing to its involvement in immune modulation and cellular recovery mechanisms.

KLRG1 was prominently associated with immune signaling and cellular machinery. KEGG analysis (Figure 9E) revealed its involvement in primary immunodeficiency, antigen processing and presentation, and T cell signaling pathways, emphasizing its role in coordinating immune responses. GO analysis (Figure 9F) demonstrated significant enrichment in ribosomal large subunit biogenesis, translational regulation, and metabolic processes, highlighting its contribution to immune cell functionality and systemic metabolic adaptations.

These findings collectively emphasize that CCR5, CYSLTR1, and KLRG1 are intricately involved in regulating immune responses, cellular metabolism, and protein turnover, providing a mechanistic basis for their diagnostic relevance in severe COVID-19.

## 4 Discussion

The onset and development of COVID-19 involve intricate gene expression changes and immune dysregulation, yet the dynamic nature of these processes remains elusive. Prior studies by Arunachalam et al. (2020), Overmyer et al. (2021), and Hocini et al. (2023) have offered valuable insights but also left notable gaps. Arunachalam et al. explored immune responses in COVID-19 patients and healthy controls, finding reduced HLA - DR and proinflammatory cytokine expression in myeloid cells, along with impaired mTOR and IFN-α-related functions in plasmacytoid dendritic cells (Arunachalam et al., 2020). However, their study did not comprehensively cover gene expression and immune regulation across all COVID-19 stages, including mild to severe cases and recovery. Overmyer et al. conducted a large-scale multi-omic analysis, identifying 219 biomolecules associated with COVID-19 severity, mainly focusing on complement activation, lipid transport, and neutrophil activation (Overmyer et al., 2021). But they lacked a detailed look at gene expression and immune cell changes during disease progression and recovery. Hocini et al. investigated immunological dysfunction in convalescent severe COVID-19 patients, revealing persistent abnormalities in immune cell phenotypes, serum biomarkers, and gene expression related to platelet and neutrophil activation (Hocini et al., 2023). Still, their research was mainly centered on the convalescent stage and did not integrate data from various disease stages.

In contrast, our study fills these gaps by integrating three transcriptomic datasets. We comprehensively analyze gene expression and immune regulation across all COVID-19 stages, from healthy to different severity levels and during recovery. This enables us to better understand the disease’s molecular mechanisms, identify potential diagnostic biomarkers, and lay a stronger foundation for developing effective therapeutic strategies.

Through multidimensional reduction analyses (PCA, UMAP, and t-SNE), we identified significant gene expression differences among the healthy, moderate, severe, and ICU patient groups. These progressively intensified expression changes reflect the dynamic molecular regulation during disease progression. In particular, the persistent upregulation of the LGALS2 gene from the moderate to severe and ICU stages suggests its potential key role in COVID-19 pathogenesis. Additionally, modular gene analysis revealed that gene functions during different stages of COVID-19 gradually shift from innate immune activation (e.g., phagocytosis and myeloid leukocyte activation) to adaptive immune activation (e.g., T-cell differentiation and signal transduction). This dynamic shift highlights the critical role of immune responses during disease progression and uncovers significant metabolic reprogramming in the later stages of the disease (e.g., enhanced mitochondrial function and increased ATP demand).

In recovery phase analyses, although the differences among the 1-month, 3-month, and 6-month groups were relatively small, certain dynamic changes in immune and metabolic pathways were still observed. The 1-month group exhibited strong antiviral defense and metabolic activity, which gradually normalized by the 3-month and 6-month stages, yet did not fully return to the baseline levels of the healthy group. Notably, in the 6-month group, adaptive immunity (e.g., T-cell differentiation and signal transduction) and organelle remodeling remained significantly active. This indicates that even after apparent clinical recovery, the molecular and immune systems of COVID-19 patients may require a longer time to fully recover.

This study found that CRP levels in ICU patients were significantly higher than those in non-ICU patients, indicating that CRP, as an acute-phase inflammatory marker, can reflect the inflammatory activation state in COVID-19 patients. CRP is secreted by the liver in response to cytokines such as IL-6 and is typically associated with disease severity and prognosis (Olson et al., 2023; Yao et al., 2019). However, the specificity of CRP is relatively low, as it cannot distinguish between inflammation caused by viral infections and other infectious or non-infectious inflammatory states (Rahali et al., 2024). This limitation reduces its utility as a standalone diagnostic marker. D-Dimer reflects significant activation of fibrin degradation and the coagulation system, and its elevation is closely associated with microvascular thrombosis, disseminated intravascular coagulation (DIC), and systemic inflammatory responses (Franchini et al., 2024). A marked increase in D-dimer levels is a characteristic feature of severe COVID-19 cases. In this study, D-dimer levels in ICU patients were significantly higher than those in non-ICU patients, consistent with previous findings (Wool and Miller, 2021; Song et al., 2021; Nemec et al., 2022; Rizal et al., 2022; Gul et al., 2023). In this study, the area under the ROC curve (AUC) for D-dimer showed good diagnostic performance, offering greater sensitivity compared to traditional scoring systems. This suggests that D-dimer has clinical value in assessing disease severity and early identification of high-risk patients with COVID-19. However, the specificity of D-dimer is also relatively low, as its elevation may result from other pathological conditions such as malignancies or trauma. Therefore, it should be used in conjunction with other markers (e.g., inflammatory markers or molecular biomarkers) for comprehensive evaluation. Future studies should explore the dynamic changes of D-dimer and its association with the pathophysiology of COVID-19, such as its link to endothelial damage and immune dysregulation. Additionally, combining gene expression data with clinical monitoring of D-dimer may provide more reliable insights for precision stratification of COVID-19 patients. SOFA (Sepsis-related Organ Failure Assessment) and APACHE II (Acute Physiology and Chronic Health Evaluation II) scores are widely used clinical tools for assessing disease severity and predicting prognosis in ICU patients. In this study, SOFA and APACHE II scores were significantly higher in ICU patients compared to non-ICU patients, indicating their utility in reflecting organ dysfunction in severe COVID-19 cases (Vincent et al., 1996). Elevated SOFA scores typically indicate the occurrence of multiple organ failure, while APACHE II scores, which are based on comprehensive evaluations of physiological and health conditions, can predict disease progression to some extent. Mohammad et al. reported that daily SOFA scores are better predictors of mortality than APACHE II in critically ill COVID-19 patients, though neither achieved high precision in predicting outcomes (Beigmohammadi et al., 2022). In this study, the AUC for SOFA was 0.607, higher than the AUC for APACHE II (0.557), aligning with previous findings. The primary limitation of these traditional scores lies in their reliance on clinical observations and biochemical indices, without reflecting the molecular mechanisms underlying COVID-19. For example, these scores do not reveal the specific molecular basis of immune dysregulation or cytokine storms. Additionally, their accuracy may be affected by individual differences in patients, such as age and comorbidities, limiting their applicability in COVID-19 severity stratification.

To overcome these limitations, this study suggests integrating molecular biomarkers such as CCR5, CYSLTR1, and KLRG1 with traditional scoring systems. The integration of molecular and clinical data could provide a more comprehensive assessment of disease severity and immune status in COVID-19 patients. Compared to traditional indices, the molecular biomarkers identified in this study offer distinct advantages. These genes provide a deeper understanding of the severity of COVID-19 from a molecular perspective, complementing traditional clinical assessments.

CCR5 is a key chemokine receptor highly expressed on T cells, monocytes, and macrophages. It plays a critical role in immune cell migration and targeted recruitment to sites of inflammation (Lin et al., 2024). Immune infiltration analysis revealed that CCR5 was significantly positively correlated with CD8^+^ T cells, resting and activated CD4^+^ memory T cells, and regulatory T cells (Tregs), further supporting its crucial role in maintaining T cell activation and adaptive immune function. In severe COVID-19 patients, suppression of adaptive immune function is a hallmark of disease progression. Studies have shown that downregulation of CCR5 may limit T cell recruitment and function, contributing to immune imbalance (Ziliotto et al., 2024; Ferrero et al., 2021). Additionally, CCR5 was negatively correlated with neutrophils and monocytes, suggesting it may help suppress excessive innate immune activation to maintain immune balance and prevent immune overactivation from damaging the host.

CYSLTR1 (Cysteinyl Leukotriene Receptor 1) is a key receptor in the leukotriene signaling pathway and plays a central role in allergic inflammatory diseases such as asthma (Zhang et al., 2006; Rabinovitch et al., 2018). CYSLTR1 is predominantly expressed in airway smooth muscle cells, mast cells, eosinophils, monocytes, and macrophages, mediating chronic airway inflammation and tissue damage through bronchoconstriction, increased vascular permeability, and inflammatory cell infiltration (Trinh et al., 2019). A study by Halef Okan Doğan and colleagues revealed dysregulated leukotriene metabolism in COVID-19 patients, with CYSLTR1 expression higher in the ICU group compared to the non-ICU group (Doğan et al., 2024). Immune infiltration analysis demonstrated that CYSLTR1 was significantly positively correlated with monocytes and moderately associated with resting NK cells and mast cells. These findings suggest that CYSLTR1 may influence the inflammatory cascade in severe COVID-19 patients by modulating monocyte-macrophage system activity. In asthma management, leukotriene receptor antagonists (e.g., montelukast) have been widely used to control inflammation and bronchoconstriction (Trinh et al., 2019). These drugs inhibit CYSLTR1 signaling, reducing leukotriene-mediated inflammation and cell recruitment. Given the abnormal expression of CYSLTR1 and its potential involvement in COVID-19-related inflammation, leukotriene receptor antagonists hold promise as adjunctive therapies for severe COVID-19 patients. However, clinical studies specific to COVID-19 are still lacking, and further research is needed to validate their efficacy in controlling inflammation and promoting tissue repair.

KLRG1 is an inhibitory receptor expressed on NK cells and a subset of T cells, including CD8^+^ T cells, and serves as a marker of NK cell maturation and terminal differentiation (Hu et al., 2018; Zhang et al., 2024). Immune infiltration analysis revealed a significant positive correlation between KLRG1 and both resting and activated NK cells, indicating its role as a molecular marker of NK cell functional status in severe COVID-19 patients. NK cells are critical effectors in the antiviral immune response during COVID-19. Elevated KLRG1 expression suggests enhanced NK cell functionality but potentially reduced proliferative capacity, which could limit the maintenance of long-term immune responses in the context of persistent viral infection. Moreover, the abnormal expression of KLRG1 may regulate other immune cells through its inhibitory signals, further contributing to immune dysfunction.

The detection of these molecular biomarkers through gene chips or liquid biopsies enables rapid diagnosis, particularly under the constraints of limited ICU resources, offering opportunities for optimized resource allocation and early identification of high-risk patients. This integrated approach, combining molecular biomarkers with traditional clinical indicators, provides a new direction for precision medicine applications in COVID-19. By integrating molecular and clinical data, this method not only enhances the accuracy of diagnosis and prognostic assessment for COVID-19 patients but also provides a scientific basis for developing personalized intervention strategies.

Although this integrated multi-omics analysis provides significant insights into the progression and recovery of COVID-19, it is important to recognize certain limitations. Unfortunately, the patient cohorts used in this study did not include documentation of vaccination histories and reinfection cases. These factors can have a notable influence on immune responses and potentially impact the performance of the identified biomarkers (Condac et al., 2024; Uno et al., 2024). Additionally, the datasets did not incorporate variant - specific stratification. This lack of stratification limits our ability to fully understand how different SARS-CoV-2 variants might modify the molecular signatures we’ve identified. Another aspect to consider is that the study utilized publicly available datasets, which come with their own set of challenges. The sample size was relatively limited, and there were issues related to data heterogeneity. As the COVID-19 pandemic has evolved and started to subside, the number of new cases has decreased substantially. This has made it increasingly difficult to obtain samples, particularly from severe cases and for long - term follow - up studies. Such limitations pose obstacles to expanding the scale of the research and validating the generalizability of our findings. Moreover, the data sourced from multiple public databases may introduce technical differences and batch effects due to variations in experimental platforms, which could potentially affect the results to some extent. To address these limitations and further advance our understanding of COVID-19, future research could focus on including comprehensive clinical metadata, such as vaccination status and information about viral variants. Additionally, integrating large - scale, multicenter cohorts and adopting a combination of retrospective and prospective study designs would be beneficial. These approaches can help validate the stability and clinical utility of the identified molecular biomarkers, ultimately leading to a more profound comprehension of the pathophysiology of COVID-19.

In conclusion, this study comprehensively analyzed the gene expression patterns and regulatory features across different stages of COVID-19 progressive and recovery stages. By integrating transcriptomic data and machine learning methods, CCR5, CYSLTR1, and KLRG1 were successfully identified as efficient diagnostic biomarkers for distinguishing ICU patients, demonstrating significantly better diagnostic performance than traditional clinical indicators. These findings not only reveal key molecular characteristics of COVID-19 progression and recovery but also provide a scientific basis for improving clinical stratification accuracy and optimizing patient management strategies. The identified biomarkers can facilitate early identification of severe cases and guide personalized interventions, advancing the application of precision medicine in COVID-19. Future studies should validate these biomarkers in larger independent cohorts and further elucidate their roles in disease progression and immune recovery, offering new directions for the development of diagnostic and therapeutic strategies for COVID-19 and similar infectious diseases.

## Data Availability

The original contributions presented in the study are included in the article/Supplementary Material, further inquiries can be directed to the corresponding author.
