# Supplementary material for: Integrated transcriptomic analysis of COVID-19 stages and recovery: insights into key gene signatures, immune features, and diagnostic biomarkers through machine learning
Source: Front Genet. 2025 May 15;16:1599867. doi: 10.3389/fgene.2025.1599867 (PMC12119500; doi:10.3389/fgene.2025.1599867)
Supplement: Supplementary file 1 [file Supplementaryfile1.docx]

**Table 1.** Information and grouping of the samples.

|  | Number of samples (included) | Platforms | Status |
| --- | --- | --- | --- |
| GSE152418 | 17 | GPL24676 Illumina NovaSeq 6000 | Healthy |
|  | 4 |  | Moderate |
|  | 8 |  | Severe |
|  | 4 |  | ICU |
| GSE227116 | 10 | [GPL16791 Illumina HiSeq 2500](https://www.ncbi.nlm.nih.gov/geo/query/acc.cgi?acc=GPL16791) | Healthy |
|  | 22 |  | 1 month hospital discharge |
|  | 25 |  | 3 month hospital discharge |
|  | 18 |  | 6 month hospital discharge |
| GSE157103 | 50 | GPL24676 Illumina NovaSeq 6000 | COVID-19&ICU |
|  | 50 |  | COVID-19 |

**Table 2.** Clinical information from 100 patients with COVID-19 of varying disease severity.

|  | **COVID-19(N=50)** | **COVID-19&ICU(N=50)** | **Total(N=100)** |
| --- | --- | --- | --- |
| **CCR5** |  |  |  |
| Mean±SD | 3.11±0.67 | 1.71±0.72 | 2.41±0.99 |
| Median[min-max] | 3.20[1.65,4.24] | 1.63[0.24,3.59] | 2.40[0.24,4.24] |
| **CYSLTR1** |  |  |  |
| Mean±SD | 4.10±0.60 | 2.97±0.75 | 3.53±0.88 |
| Median[min-max] | 4.21[2.68,5.26] | 3.16[1.46,4.72] | 3.63[1.46,5.26] |
| **KLRG1** |  |  |  |
| Mean±SD | 2.55±0.79 | 1.20±0.63 | 1.88±0.98 |
| Median[min-max] | 2.64[0.67,3.86] | 1.15[0.30,2.92] | 1.83[0.30,3.86] |
| **CRP Level (mg/L)** |  |  |  |
| Mean±SD | 119.82±95.49 | 158.72±107.97 | 140.54±103.62 |
| Median[min-max] | 114.00[1.00,430.50] | 147.30[2.80,408.80] | 128.20[1.00,430.50] |
| **SOFA Score** |  |  |  |
| Mean±SD | 6.50±3.62 | 8.30±4.05 | 8.11±4.01 |
| Median[min-max] | 7.00[2.00,12.00] | 7.50[2.00,19.00] | 7.00[2.00,19.00] |
| **APACHE II Score** |  |  |  |
| Mean±SD | 19.71±5.91 | 21.58±8.49 | 21.35±8.19 |
| Median[min-max] | 19.00[11.00,27.00] | 21.50[6.00,43.00] | 21.00[6.00,43.00] |
| **Lactate Level (mmol/L)** |  |  |  |
| Mean±SD | 1.19±0.53 | 1.27±0.49 | 1.24±0.51 |
| Median[min-max] | 1.09[0.65,3.28] | 1.20[0.50,2.85] | 1.17[0.50,3.28] |
| **Procalcitonin Level (ng/mL)** |  |  |  |
| Mean±SD | 1.71±5.82 | 4.43±12.89 | 3.24±10.45 |
| Median[min-max] | 0.36[0.05,36.00] | 1.02[0.05,86.39] | 0.57[0.05,86.39] |
| **Age (years)** |  |  |  |
| Mean±SD | 58.96±18.40 | 62.64±13.60 | 60.84±16.15 |
| Median[min-max] | 56.50[24.00,87.00] | 63.00[21.00,83.00] | 62.00[21.00,87.00] |
| **D-dimer Level (mg/L FEU)** |  |  |  |
| Mean±SD | 1.98±3.21 | 18.88±27.52 | 11.72±22.53 |
| Median[min-max] | 0.99[0.22,15.48] | 6.03[0.59,104.42] | 1.79[0.22,104.42] |
| **Sex** |  |  |  |
| female | 21(21.00%) | 17(17.00%) | 38(38.00%) |
| male | 29(29.00%) | 33(33.00%) | 62(62.00%) |
| **Fibrinogen Level (g/L)** |  |  |  |
| Mean±SD | 563.56±191.72 | 528.96±201.64 | 543.85±196.94 |
| Median[min-max] | 513.00[215.00,949.00] | 490.00[140.00,949.00] | 513.00[140.00,949.00] |
| **Charlson Comorbidity Index** |  |  |  |
| Mean±SD | 3.12±2.46 | 3.44±2.51 | 3.28±2.48 |
| Median[min-max] | 2.50[0,8.00] | 3.00[0.0,11.00] | 3.00[0,11.00] |
